# Advances of MXene in Detection and Sterilization of Foodborne Pathogens

**DOI:** 10.3390/foods14223807

**Published:** 2025-11-07

**Authors:** Wenjie Gao, Han Yan, Rui Wang, Wei Wu, Qinzhi Wang

**Affiliations:** 1 College of Food Science and Engineering, Qingdao Agricultural University, Qingdao 266109, China; 18364876682@163.com (W.G.); yanhanqau@163.com (H.Y.); 20242117095@stu.qau.edu.cn (R.W.); 2 Shandong Technology Innovation Center of Special Food, Qingdao 266109, China; 3 Qingdao Institute of Special Food, Qingdao 266109, China

**Keywords:** MXene, foodborne pathogens, detection, sterilization

## Abstract

MXene, owing to its high electrical conductivity, large specific surface area, and abundant surface functional groups, has been widely applied in the detection of foodborne pathogens. Therefore, it is necessary to review recent developments in the emerging material MXene for the detection and killing of foodborne pathogens, which is expected to facilitate the further development and utilization of MXene. This work comprehensively reviews advances in MXene applications for detecting and killing foodborne pathogens. Firstly, applications of MXene in electrochemical sensors, surface-enhanced Raman scattering (SERS), fluorescence platforms, and fluorescence–electrochemical dual-mode sensing systems are introduced. Subsequently, the sterilization mechanisms of MXene are described, followed by a detailed explanation of its practical applications in active food packaging, surface modification of food processing equipment, and instant sterilization techniques. Finally, conclusions, challenges, and future prospects in the area of MXene for the detection and killing of foodborne pathogens are discussed in depth. Significantly, this review uniquely summarizes applications of MXene in the detection and sterilization of foodborne pathogens, offering new perspectives on its use in food safety.

## 1. Introduction

Foodborne pathogens are microorganisms that cause diseases through contaminated food, leading to acute symptoms such as vomiting and diarrhea. In severe cases, they can even cause sepsis, meningitis, or death, posing significant risks to public health. Common key bacterial species that cause human foodborne illness through their pathogenic effects include *Bacillus cereus*, *Listeria monocytogenes*, *Salmonella* species, and *Staphylococcus aureus*, among others [1,2,3,4,5,6,7]. According to the World Health Organization, foodborne pathogens cause approximately 600 million cases of illness and 420,000 deaths globally each year, including 125,000 children under the age of five. In the United States alone, the economic losses amount to USD 17 billion. Among the major pathogens, “Salmonella” causes 155 million cases annually, “Campylobacter” causes 96 million cases, and “Listeria”, though it has fewer cases (23,000), has a mortality rate as high as 30%. More alarmingly, about 600,000 deaths are linked to antibiotic resistance, with multi-drug-resistant strains increasing treatment costs by 3–5 times. This public health crisis is particularly acute in developing countries, underscoring the urgent need to strengthen global food safety systems. To safeguard public health, more accurate and rapid methods are required for the efficient detection and sterilization of foodborne pathogens.

The prevention and control of foodborne pathogens can be divided into two aspects: detection and sterilization. In terms of detection, traditional methods for detecting foodborne pathogens include the plate counting method, PCR, ELISA, and IMS [7,8,9,10,11,12,13,14,15]. These methods are characterized by their simplicity of operation, low cost, and reliable results, making them fundamental tools for foodborne pathogen detection. In recent years, new rapid detection technologies for foodborne pathogens have garnered widespread attention and application due to their advantages of fast detection speed, simple pre-treatment, and high sensitivity and efficiency [7,12,14,16,17,18]. Generally, various nanomaterials play significant roles in constructing rapid detection technologies, which can dramatically amplify sensing signals. MXene is a new class of two-dimensional materials [19,20,21,22,23,24,25,26,27], composed of transition metal carbides, nitrides, or carbonitrides with the chemical formula M_n+1_X_n_T_x_ [28,29,30,31,32], where M is a transition metal such as Ti or Mo, X is C/N, and T_x_ is a surface functional group such as –O, –F or –OH [33,34]. It is prepared by the selective etching of the Al layer in MAX phases and has atomic-level thickness, high electrical conductivity (≈6000 S/cm), rich surface chemical properties, and tunable electronic structure [24]. The hydrophilicity and large specific surface area of MXene make it easy to disperse and functionalize. Owing to its metal-like conductivity, large specific surface area, abundant surface functional groups (–OH, –F, =O) [35], excellent hydrophilicity, mechanical flexibility, and tunable bandgap [36], MXene (typically Ti_3_C_2_T_x_) has rapidly emerged as a “star material” in cutting-edge applications in the detection of foodborne pathogens. For example, Duan et al. [37] developed a novel electrochemical biosensor based on CG@MXene nanocomposites and CRISPR/Cas12a (E-CRISPR) for the rapid and sensitive detection of *Salmonella typhimurium*. Due to the excellent functionalizable modification properties of MXene in the prepared CG@MXene nanocomposite, the electrode surface can be modified to reduce the initial signal (background noise) of the electrode, thereby improving detection sensitivity. Liu et al. [38] constructed a dual-mode photoelectrochemical and surface-enhanced Raman scattering biosensor based on carbon nitride nanosheets (C_3_N_4_)/MXene-gold nanoparticle accelerators. The two-dimensional layered structure of MXene provides a uniform carrier. This carrier supports the in situ growth of gold nanoparticles (Au NPs). The uniform carrier prevents Au NPs from agglomerating. High-density plasmonic “hot spots” are formed. These “hot spots” synergistically enhance the SERS signal. A dual-recognition binding-induced DNA walker is combined with this system. This combination enables highly sensitive detection. It also ensures accurate detection of Staphylococcus aureus. When compared to other 2D nanomaterials like graphene oxide, MXene offers superior hydrophilicity and richer surface chemistry, which facilitates biomolecule immobilization. However, its environmental stability, particularly under humid conditions, remains a challenge that needs to be addressed in practical applications. Additionally, due to the physical cutting action of its sharp edges, reactive oxygen species (ROS) generation is induced by surface metal ions, and localized high temperatures (>50 °C) are produced under near-infrared (NIR) light irradiation [39,40,41]. MXene exhibits remarkable antibacterial effects, leading to bacterial protein denaturation and membrane rupture [39,42,43,44]. For instance, Huang et al. [45] developed a novel Z-scheme heterojunction material, MXene/TiO_2_/Bi_2_S_3_, which generates ROS through photothermal catalysis. The presence of MXene significantly enhances ROS production efficiency, resulting in complete inactivation of both *Escherichia coli* and *Staphylococcus aureus*. Ahmad Arabi Shamsabadi et al. [43] demonstrated that smaller Ti_3_C_2_ nanosheets exhibit significant antibacterial activity by inducing membrane structural damage through direct physical contact between their two-dimensional sheet edges and bacterial cell membranes. Therefore, the rational design and preparation of MXene and its composites can play an important role in the detection, prevention, and control of foodborne pathogens.

**Figure 1:**
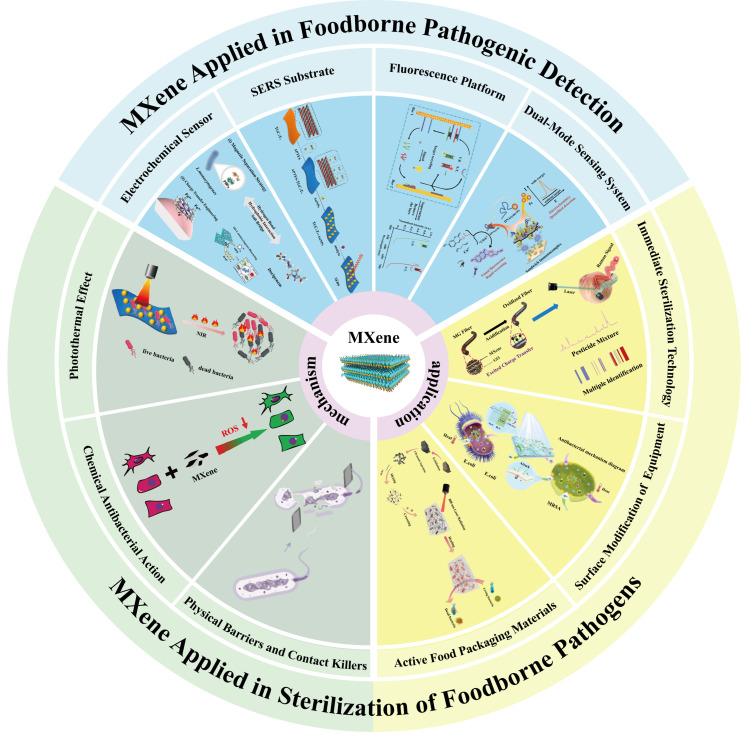
Application of MXene in detecting and sterilization foodborne pathogens.

Although MXenes have been successfully applied to the control of foodborne pathogens, there have been few systematic summaries on the detection and elimination of foodborne pathogens by MXene up to now. In this work, the progress in MXene applications in the detection and sterilization of foodborne pathogens is systematically discussed (Figure 1). The serious threat posed by foodborne pathogens to global public health is emphasized, and the limitations of traditional detection and sterilization methods are pointed out. Special attention is paid to the unique properties of MXene that render it an ideal material for efficient detection and sterilization of foodborne pathogens. Finally, practical application cases of MXene in detection and sterilization technologies are comprehensively reviewed, indicating that further research needs to be conducted to explore its biosafety, large-scale production, and multi-mode synergistic mechanisms. Despite numerous studies on MXene-based biosensors and antibacterial composites, a consolidated understanding of their integrated role in foodborne pathogens control remains limited. This review aims to bridge that gap.

## 2. MXene Applied in Foodborne Pathogen Detection

MXene has high electrical conductivity, a large specific surface area, abundant surface functional groups, and a tunable electronic structure [20,46,47,48,49,50,51,52,53]. Thanks to these characteristic structures, MXene can be used as an ideal platform for the construction of biosensors in the detection of foodborne pathogens, including electrochemical sensors, SERS substrates, fluorescence platforms, and dual-mode sensing systems (Table 1).

**Table 1:** Performance of MXene-based sensors in the detection of foodborne pathogens.

Material-Based Substrate	Target Bacteria	Limit of Detection (LOD) (CFU/mL)	Linear Detection Range (CFU/mL)	Recovery (%)	Relative Standard Deviation (RSD) (%)	R^2^	References
CG@MXene	*S. Typhimurium*	160	1.6 × 10^2^–1.6 × 10^7^	100.46–106.37	4.56–7.28	0.99	[37]
MXene-Hemin-Au@mAb-based biosensor	*L. monocytogenes*	23	10–10^6^	91.19–102.98	-	0.991	[54]
PDA@ZnMoO_4_/MXen	*L. monocytogenes*	12	10–10^7^	98–126	1.63–5.85	0.991	[55]
DNAzyme-Fc-MXene@AuNBPs	*V. parahaemolyticus*	6	10–10^8^	94.0–106.0	4.26–8.51	0.995	[56]
LAP-MXene@AuNPs-ssDNA MRS	*V. parahaemolyticus*	10	10^2^–10^8^	97.4–103.2	1.8–5.6	0.998	[57]
cDNA-POSS-PQDs	*V. parahaemolyticus*	10	10^2^–10^6^	93–108	1.9–7.4	0.9981	[58]
MXene/polypyrrole	*Salmonella*	23	10^3^–10^7^	96–109.4	1.33–2.87	-	[59]
Ag@TiO_2_/MXene	*E. coli O157:H7*	1	1–17	-	-	-	[60]
MXene@MB	*Salmonella*	5	2.4 × 10^1^–2.4 × 10^7^	98.3–102.2	-	0.9958	[61]

### 2.1. Application of MXene-Based Electrochemical Sensor in the Detection of Foodborne Pathogens

The application mechanisms of MXene in electrochemical detection mainly include the following aspects: (1) it enhances signal response by virtue of high electrical conductivity; it strengthens biomolecular recognition relying on large specific surface area and active functional groups [62,63,64,65]. (2) It gains catalytic activity through regulating the electronic structure to amplify signals [66,67]. (3) It uses surface functional groups to flexibly immobilize bio-recognition elements and has good biocompatibility. (4) It produces a synergistic effect when compounded with other materials to further improve performance [68,69,70,71,72,73,74,75,76,77,78]. Overall, through the construction of conductive networks, regulation of catalytic activity, and optimization of interface engineering, MXene exhibits highly sensitive and selective performance in electrochemical detection. In MXene-based electrochemical biosensing research, significant progress has been made in the highly sensitive detection of foodborne pathogens.

**Figure 2:**
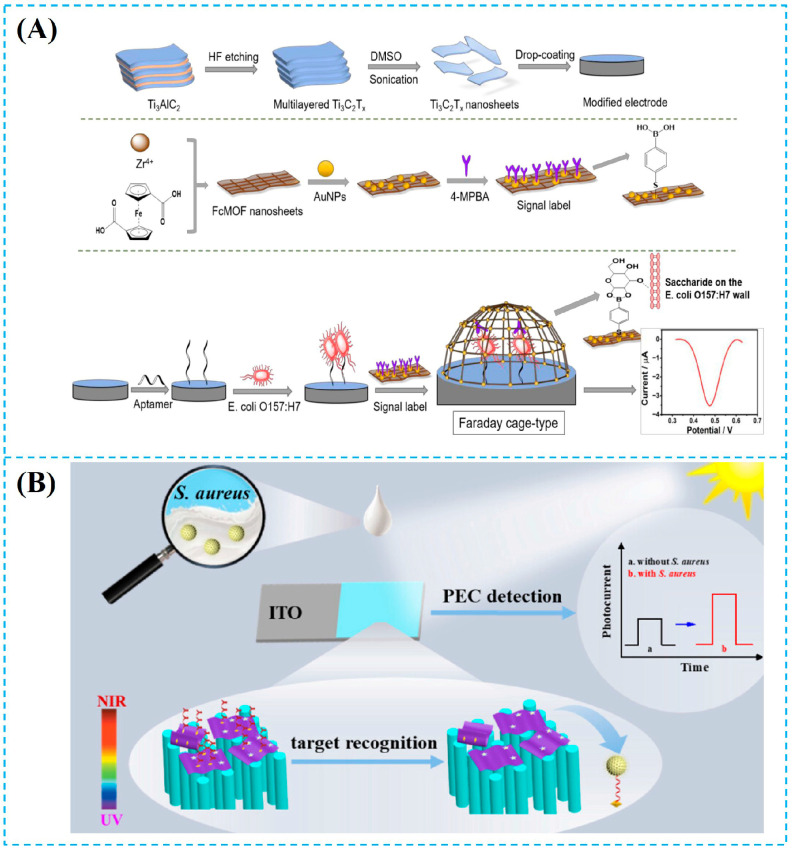
(**A**) The mechanism of the aptasensor involves the synthesis of Ti_3_C_2_T_x_ and electrode modification, the synthesis of Zr-Fc-MOF/AuNPs/4-MPBA signal labels, and a schematic diagram for the detection of *Escherichia coli O157:H7* (Zr-Fc-MOF: Zirconium-based Ferrocene-labeled metal–organic framework; AuNPs: gold nanoparticles; 4-MPBA: 4-Mercaptophenylboronic Acid) [79]. (**B**) Mechanism schematic of the PEC aptasensor for *Staphylococcus aureus* detection (PEC: photoelectrochemical) [80]. Reproduced with permission from Refs. [79,80]: Dai et al. (2022) and Huang et al. (2024).

Li et al. [54] have developed excellent sensing systems based on MXene for the detection of *Listeria monocytogenes* (*L. monocytogenes*) (Figure 2A). MXene (Ti_3_C_2_) is used as a carrier and electronic regulation platform for the construction of a MXene–Hemin–Au composite nanozyme. Through charge transfer engineering, MXene regulates the valence state conversion between Fe^3+^ and Fe^2+^ in Hemin, significantly enhancing its peroxidase-like (POD) activity. Then, the TMB/H_2_O_2_ can be catalyzed to generate electrochemical signals. Combined with the efficient capture of magnetic microspheres modified by the antibiotic doripenem, this sensing system achieves highly sensitive and quantitative detection of the *L. monocytogenes* with the detection limit of 23 CFU/mL. Baskaran et al. [55] used a polydopamine (PDA)-modified ZnMoO_4_/MXene composite material to modify the electrode and designed a label-free electrochemical immunosensor for rapid detection of *Listeria monocytogenes* (Figure 2B). MXene (Ti_3_C_2_T_x_) works synergistically with 3D flower-like ZnMoO_4_ and PDA to form a modified layer with high conductivity and a large specific surface area. The PDA layer directly immobilizes antibodies to achieve highly selective recognition of *L. monocytogenes*. The sensor shows a good linear relationship in the range of 10–10^7^ CFU/mL with a detection limit of 12 CFU/mL. In this system, MXene effectively inhibits the agglomeration of ZnMoO_4_ and enhances electron transport efficiency (electrochemical impedance spectroscopy shows a decrease in charge transfer resistance Rct). MXene also works with the adhesiveness of PDA and the catalytic activity of ZnMoO_4_ to further improve detection sensitivity. The above studies indicated the MXene can be successfully used in electrochemical sensors, which achieves highly sensitive and selective detection of foodborne pathogens through structural design and performance regulation with good performance. Beyond electrochemical sensing, MXene’s unique optical and electronic properties have also enabled its application as a high-performance substrate for SERS, which will be discussed in the next section.

### 2.2. Application of MXene-Based SERS Substrate in the Detection of Foodborne Pathogens

**Figure 3:**
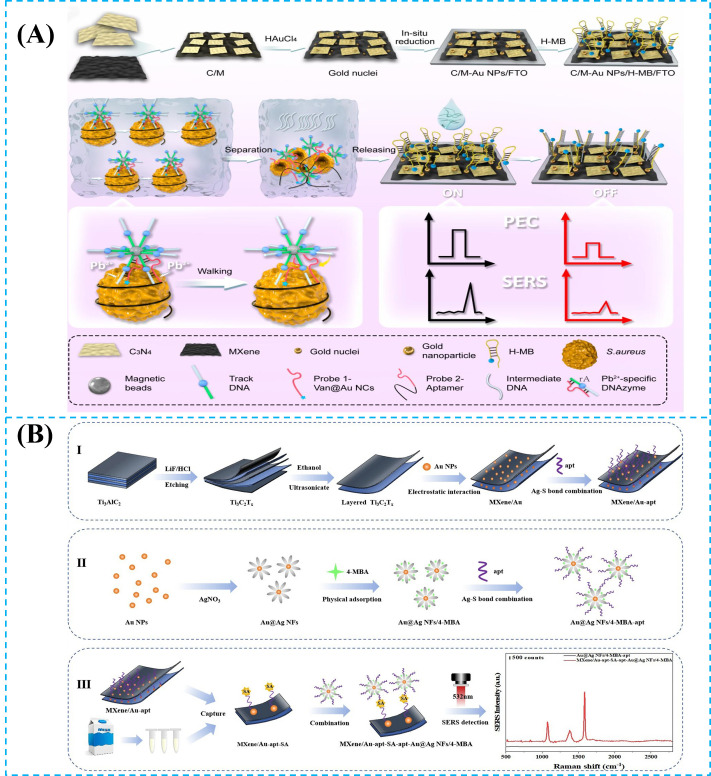
(**A**) C/M-gold nanoparticles (C/M-Au NP_S_) were synthesized, which were combined with the DNA walker for the signal amplification strategy, leading to sensitive and accurate PEC-SERS dual-mode detection of *S. aureus* (PEC: photoelectrochemical; SERS: surface-enhanced Raman scattering; C_3_N_4_: carbon nitride; Au NPs: gold nanoparticles) [38]. (**B**) (**Ⅰ**) Schematic diagram of the preparation of MXene/Au-aptamer composite material. (**Ⅱ**) Schematic diagram of the preparation of Au@Ag NPs/4-MBA-aptamer signal probe. (**Ⅲ**) Schematic diagram of the SERS detection principle for *S. aureus*. (Au@Ag NFs: gold-core silver-shell nanoflowers; 4-MBA: 4-Mercaptobenzoic Acid; SERS: surface-enhanced Raman scattering) [21]. Reproduced with permission from Refs. [38,21]: Liu et al. (2023) and Qu et al. (2025).

MXene acts as a multifunctional synergistic amplification platform in SERS systems [81,82,83,84,85,86,87,88,89]. MXene has a highly conductive 2D framework (≈10^4^ S cm^−1^), which allows uniform anchoring of 10–15 nm plasmonic metal nanoparticles [21]. The localized surface plasmon resonance (LSPR) electromagnetic hotspots are efficiently coupled to the external circuit, which prevents charge accumulation quenching. MXene enables precise enrichment and orientation of probe molecules through hydrogen bonds, electrostatic interactions, or π-π stacking, which ensures molecules are positioned in the plasmonic metal nanoparticles. Furthermore, a Schottky junction forms between MXene and Au, which allows directional electron injection from MXene into plasmonic metal nanoparticles under light excitation. The charge transfer barrier between the metal and molecules is reduced, which contributes a 10^2^–10^3^-fold chemical enhancement. Combined with electromagnetic enhancement, the total enhancement factor can reach 10^8^. Based on this mechanism, Liu et al. [38] developed a photoelectrochemical (PEC) surface-enhanced Raman scattering (SERS) dual-mode biosensor based on a C_3_N_4_/MXene-AuNP accelerator, which achieves highly sensitive detection of Staphylococcus aureus through a dual-recognition DNA walker mechanism. In Figure 3A, the preparation of the dual-mode sensor and the process through which it can be integrated into the induced DNA walker to achieve the detection of Staphylococcus aureus are introduced. The negatively charged MXene surface enables the electrostatic self-assembly of 12 nm Au NPs, which provide a layered conductive network that can reduce interfacial charge transfer resistance, providing high-density SERS hotspots. The biosensor has a linear range of 5–10^8^ CFU/mL and a limit of detection (LOD) of 0.70 CFU/mL in the PEC mode and a linear range of 10–10^8^ CFU/mL and an LOD of 1.35 CFU/mL in the SERS mode. Moreover, the sensor exhibits excellent performance (with a recovery rate of 91–115%) in real samples such as milk, orange juice, and peach juice, while also possessing a strong anti-interference ability, stability, and reproducibility. In summary, MXene plays a key role in photoelectric conversion, hotspot construction, and signal amplification in the construction of SERS substrates, which provides a new strategy for on-site food safety detection. While SERS leverages MXene plasmonic enhancement capabilities, its excellent fluorescence quenching ability and large surface area also make it an ideal platform for constructing fluorescent biosensors, as detailed below.

### 2.3. Application of MXene-Based Fluorescence Platform for the Detection of Foodborne Pathogens

In MXene-mediated fluorescence detection systems, its dual functions as a “reversible quencher” and a “fixation/release platform” have been effectively verified. Liu et al. [58] developed a fluorescent aptasensor using multicolor perovskite quantum dot (CsPbX3 PQDs)-encoded DNA probes immobilized on MXene with dual stir bar-assisted signal amplification, achieving on-site multiplex detection of *viable Salmonella (S.T.*) and *Vibrio parahaemolyticus* (*V.P.*) (Figure 3B). MXene efficiently adsorbs and quench the fluorescence of perovskite quantum dots on the stir bar, which is achieved through π-π stacking and electrostatic interactions. A stable “MXene-cDNA-PQDs” composite probe is formed, which significantly reduces background signal interference. When the target viable bacteria specifically bind to the corresponding aptamer, a competitive displacement occurs in the aptamer-cDNA double strand, causing PQDs to desorb from the MXene surface, and the fluorescence is restored. The MXene-based sensor shows high sensitivity with the low detection limits of *Vibrio parahaemolyticus* and *Salmonella* as 10 CFU/mL and 30 CFU/mL, respectively, which is suitable for rapid and simultaneous detection of multiple viable pathogens in complex water samples. This study fully reflects the core value of MXene, which acts as a “reversible quencher” to regulate the conversion of fluorescent signals. At the same time, MXene serves as a “fixation/release platform” to optimize probe loading and response efficiency, which provides an important reference for the design of microbial fluorescence sensing systems. In addition to standalone fluorescence platforms, the integration of MXene into dual-mode sensing systems—combining fluorescence with electrochemical detection—has emerged as a powerful strategy to enhance detection reliability and accuracy, as explored in the following section.

### 2.4. Application of Mxene-Based Dual-Mode Sensing System in the Detection of Foodborne Pathogens

MXene simultaneously functions as a “switch controller” for fluorescent signals and an “amplifier” for electrochemical signals, enabling synchronous dual-mode signal output through a unified platform. This integrated design allows cross-validation between the two detection modes, effectively reducing false positives/negatives and significantly enhancing detection reliability. Wang et al. [56] developed a fluorescence–electrochemical dual-mode biosensor for rapid detection of *V.P.* MXene, with its ultra-large specific surface area and abundant surface functional groups, efficiently loaded gold nanobipyramids (AuNBPs) through electrostatic interactions to form a MXene@AuNBPs composite structure. This composite further immobilized antimicrobial peptides (AMPs) and ferrocene-labeled DNAzyme (DNAzyme-Fc) via Au-S/Au-NH_2_ bonds, densely integrating “recognition–catalysis–electroactivity” functional units to provide sufficient sources for dual-mode signals. Secondly, the excellent electron conductivity of MXene synergized with the plasmonic effect of AuNBPs, significantly reducing the charge transfer resistance at the electrode interface and amplifying the electrochemical signal by approximately 6.5 times, laying the foundation for accurate quantification of the target bacteria (with a detection limit as low as 6 CFU/mL). Then, the highly loaded DNAzyme in MXene@AuNBPs efficiently catalyzed the click chemical reaction (CuAAC) in the presence of Cu^2+^, driving the cycloaddition reaction between 3-azido-7-hydroxycoumarin (AHC) and 3-butyn-1-ol (BOL) to generate a strong fluorescent product, enabling rapid visual screening. Additionally, the hydrophilic surface functional groups (–OH/=O) of MXene stably bound to AMP through hydrogen bonds and electrostatic interactions, both enhancing the specific recognition of *V.P*. and reducing non-specific adsorption, ensuring the reliability of fluorescence and electrochemical signals. This MXene-based sensor combines target bacteria with signal probes via an immune sandwich reaction, provides the wide detection linear range of 10–10^8^ CFU/mL, and has been successfully applied in the detection of actual samples such as shrimp, crab, and fish. The successful implementation of MXene in various detection modalities underscores its versatility and potential as a multifunctional nanomaterial for next-generation biosensing platforms (Table 2). Beyond detection, MXene also exhibits remarkable antibacterial properties, which will be systematically discussed in the subsequent section on sterilization mechanisms and applications.

**Table 2:** Performance comparison of MXene-based sensors and other nanomaterial-based sensors for foodborne pathogen detection.

Detection Method	Materials	Target Bacteria	Limit of Detection (LOD) (CFU/mL)	Linear Detection Range (CFU/mL)	References
Electrochemical detection	Ag@TiO_2_/MXene	*E. coli O157:H7*	1	1–17	[60]
Electrochemical detection	MXene@MB	*Salmonella*	5	2.4 × 10^1^–2.4 × 10^7^	[61]
SERS	HfTe_2_-Au	*Salmonella*	10		[90]
Fluorescence platform	GO-QD	*L. monocytogenes*	100	10^2^–10^6^	[91]
Chemiluminescence biosensing platform	HRP-Ab-CaHPO4	*Salmonella enteritidis*	10	10–10^5^	[92]
Electrochemical detection	rGO-CNT	*S. Typhimurium*	10	10–10^8^	[93]
SERS	rGOPE/AuNP_S_	*E. coli O157:H7*	150	1.5 × 10^2^–1.5 × 10^7^	[94]
Electrochemical detection	ssDNA-Au/CuMOF	*S. aureus*	5	10–108	[95]

## 3. MXene Applied in Sterilization of Foodborne Pathogens

### 3.1. Antibacterial Mechanism

#### 3.1.1. Photothermal Effect

**Figure 4:**
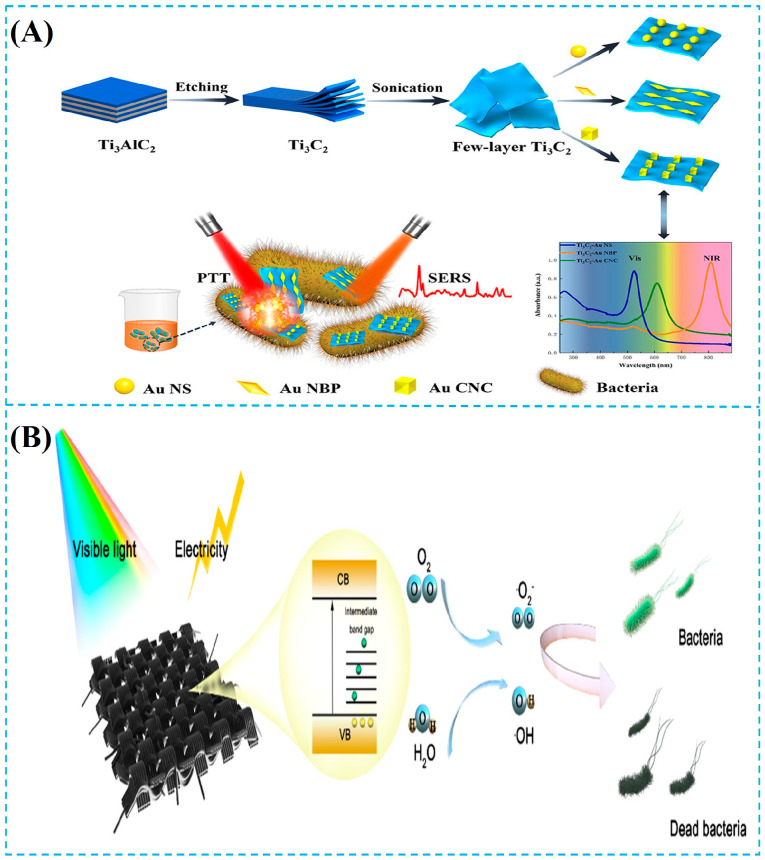
(**A**) Schematic illustration of the preparation of the Ti_3_C_2_-Au nanomaterial substrate and its application in SERS detection and NIR-triggered PTT of bacteria (SERS: surface-enhanced Raman scattering; Au NS: gold nanostar; NIR: near-infrared; PTT: photothermal therapy) [104]. (**B**) Schematic diagram of the antibacterial mechanism of B-TiO_2_–_X_NW_S_ [105]. Reproduced with permission from Refs. [104,105]: Jiang et al. (2023) and Zhang et al. (2022).

The thermal–harmonic effect [96] refers to the phenomenon where materials absorb light energy and convert it into thermal energy through non-radiative relaxation processes, causing a local temperature increase and changes in material properties. The microscopic process begins with the interaction between light waves and the material. The electrons within the material absorb the energy of photons and transition to the excited state. Subsequently, they release energy through non-radiative pathways such as vibration relaxation and phonon scattering, ultimately dissipating as heat, resulting in a significant increase in the system temperature and triggering physical and chemical changes [97]. In the field of foodborne pathogen detection and control, the photothermal effect demonstrates dual application values: post-detection sterilization and active sterilization [44,98,99,100,101]. This sterilization mechanism has the advantages of high selectivity, no chemical residues, and rapid action. The efficient photothermal converter (PTA) is at the core of this technology. Zheng et al. [102] constructed a Ti_3_C_2_ MXene-based hybrid hydrogel for highly efficient capture and eradication of methicillin-resistant *Staphylococcus aureus* (MRSA) (Figure 4A). The MXenes exhibit strong absorption within the biological transparency window (700–1100 nm), enabling localized hyperthermia (>50 °C) that disrupts bacterial membrane integrity and protein function. The integration of MXene into photothermal agents (PTAs) endows the system with low biotoxicity, high photothermal conversion efficiency, and excellent stability. As a result, the Cip-Ti_3_C_2_ nanocomposites achieved a remarkable in vitro bactericidal efficiency of >99.99999% (7.03 log_10_) against MRSA. It is worth noting that the photothermal effect often forms a synergistic mechanism with other antibacterial strategies: Xi et al. [103] designed a Ru@Bi_2_S_3_/Nb_2_C MXene Schottky heterojunction-based photocatalyst based on work function engineering, which significantly enhanced the eradication efficiency against *E. coli* and MRSA under NIR laser irradiation (Figure 4B). When combined with photodynamic therapy (PDT), the photothermal heating generated by Ru@Bi_2_S_3_/Nb_2_C accelerated the production of reactive ROS and increased bacterial membrane permeability, thereby improving the oxidative damage efficiency of PDT. In the sterilization process in the food industry, the photothermal effect also plays a crucial role. Pulsed-light technology, through the synergy of the ultraviolet spectrum (especially UV-C) and thermal effects, directly damages the DNA, proteins, and cell membrane structure of microorganisms and efficiently inactivates foodborne pathogen bacteria and their spores. In the food sterilization process, the interaction between photothermal sterilization and chemical sterilization can significantly enhance the sterilization efficiency.

#### 3.1.2. Chemical Antibacterial Action

**Figure 5:**
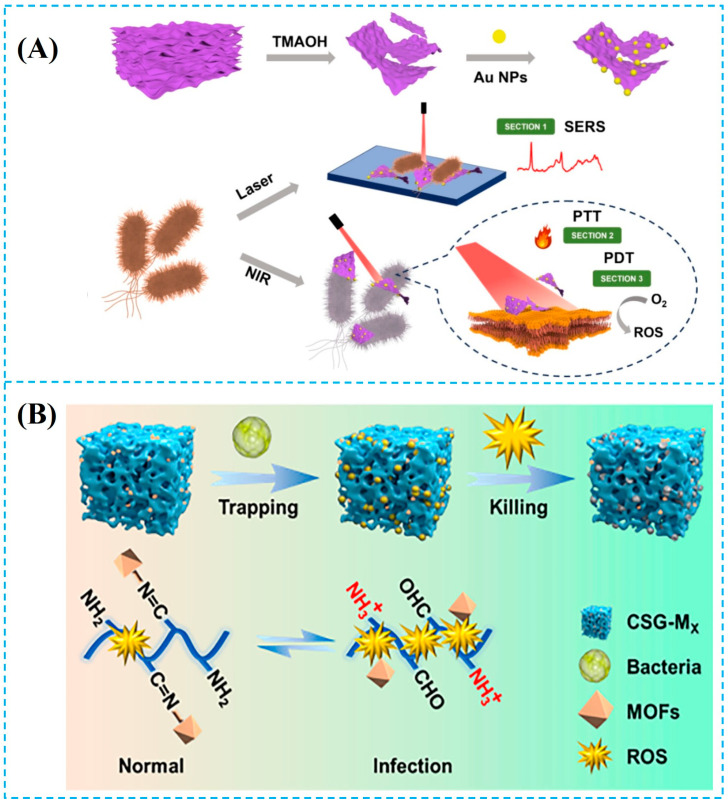
(**A**) Schematic illustration showing the process from the preparation of V_2_CT_X_-Au to its application for bacterial SERS detection and synergistic photothermal/ROS sterilization mechanism (SERS: surface-enhanced Raman scattering; PTT: photothermal therapy; PDT: photodynamic therapy; ROS: reactive oxygen species) [113]. (**B**) Schematic diagram of the sterilization mechanism of the MOF nanozyme composite cryogel (MOF: metal–organic framework) [114]. Reproduced with permission from Refs. [113,114]: Zhao et al. (2024) and Li et al. (2021).

The chemical antibacterial effect of MXene composite materials is mainly achieved through multiple pathways such as metal ion release, surface functional group action, and ROS generation [99,106,107,108]. There is a significant synergistic effect among these mechanisms. MXene composite materials often exert antibacterial effects by loading metal ions (such as Zn^2+^, Ag^+^). These metal ions bind to the negatively charged bacterial cell membrane through electrostatic attraction, disrupting the permeability of the membrane structure; after entering the bacterial body, they can further interfere with the metabolic process. For example, when Zn^2+^ is embedded in the hydrogel system, it not only inhibits bacterial ATP synthesis and amino acid metabolism but also enhances the local heat conduction efficiency, promoting the close transmission of heat energy in photothermal treatment, achieving a synergistic chemical and photothermal sterilization effect [109]. In addition, the abundant functional groups (–OH, –F) on the MXene surface can disrupt bacterial metabolism by inducing oxidative stress responses, thereby further enhancing the synergistic effect of chemical antibacterial activity. Liu et al. [110] designed a work function theory-based MXene composite (Ti_3_C_2_T_x_/TiO_2_), which achieved highly efficient sterilization against *Staphylococcus aureus* and *Salmonella typhimurium* (Figure 5A). It is possible to tune the work function of MXene through the selection of transition metals, X elements, and surface terminations. Under visible-light irradiation, the built-in electric field (IEF) formed within the material due to the work function difference drives the directional transfer of photogenerated electrons from TiO_2_ to Ti_3_C_2_T_x_. These electrons react with oxygen molecules on the surface of Ti_3_C_2_T_x_ to form superoxide anion radicals (·O_2_^−^), while the holes interact with water molecules in the valence band of TiO_2_ to generate hydroxyl radicals (·OH). These ROS exhibit strong oxidizing properties, enabling them to directly attack bacterial cell membranes, resulting in abnormal membrane permeability, protein leakage, and disruption of membrane potential stability. They can also oxidize bacterial macromolecules such as lipids, proteins, and DNA, leading to highly efficient sterilization. Meanwhile, such materials significantly inhibit bacterial coagulase activity, prevent rabbit plasma coagulation, and suppress the expression of bacterial virulence factors, thereby further enhancing the chemical antibacterial efficacy. By constructing Schottky heterojunctions (such as Ru@Bi_2_S_3_/Nb_2_C), the coupling of MXene with semiconductor materials can further optimize the efficiency of ROS generation. The built-in electric field formed by the heterojunction can effectively promote the separation of photogenerated carriers, reduce electron–hole recombination, and significantly increase the yield of ROS such as ·O_2_^−^ and ·OH. Its killing rate against drug-resistant bacteria (such as MRSA) can exceed 99.87% [103]. Tang et al. [111] prepared a superhydrophobic sandwich-structured composite film (FMX_5_-T) by modifying MXene with perfluorosilane (PFOTS), which enables controllable motion (such as linear and rotational movement) under light irradiation and achieves a 100% sterilization rate against *E. coli* and *S. aureus* (Figure 5B). Under NIR light irradiation, MXene rapidly generates heat and produces ROS, which synergize with its superhydrophobic surface to inhibit bacterial adhesion and achieve highly efficient bacterial elimination. Moreover, MXene nanosheets modified with copolymer of menthol (BPM) can increase the contact probability by specifically targeting bacterial cell membranes under near-infrared light irradiation. Their sharp edges will hinder electron transfer in the bacterial respiratory chain, leading to more electron leakage and reaction with oxygen molecules to generate ROS. At the same time, the photothermal effect accelerates the rate of intracellular biochemical reactions in bacteria, further increasing the level of ROS, intensifying the oxidative damage to bacterial biomacromolecules, and ultimately achieving efficient sterilization [112].

#### 3.1.3. Physical Barriers and Contact Killers

Not only does the antibacterial mechanism of MXene involve chemical action, but the physical barrier and contact killing also play key roles [43,115,116,117,118,119,120]. Its two-dimensional layered structure can form a dense physical barrier through van der Waals forces, effectively blocking the migration and nutrient uptake of bacteria and inhibiting the proliferation of colonies. The high specific surface area and surface negative charge characteristics (with a Zeta potential often lower than −30 mV) can enhance electrostatic adsorption with positively charged bacterial membranes, promoting the close encapsulation of MXene nanosheets around the bacteria. What is particularly important is that the atomic-level sharp structures at the edges of the nanosheets can directly physically cut the bacterial cell membranes and the matrix of biofilms, causing leakage of intracellular substances through mechanical damage [111]. Additionally, the superhydrophobic MXene composite film (such as FMX_5_-T) significantly reduces bacterial initial adhesion by lowering the surface energy (contact angle > 150°), combined with near-infrared light-triggered high-temperature hyperthermia, forming an “anti-adhesion–thermal ablation” dual physical defense system, which collaboratively enhances the blocking and killing efficiency of foodborne pathogen bacteria. While the photothermal and ROS-generating capabilities of MXene are promising for sterilization, the potential cytotoxicity and long-term food safety implications of MXene residue require thorough investigation before widespread commercial use is possible.

### 3.2. Application of MXene in Sterilization of Foodborne Pathogens

#### 3.2.1. Active Food Packaging Materials

Current antimicrobial food packaging is advancing towards green efficiency and intelligent proactivity, integrating natural biodegradable materials with nanotechnology (AgNPs, MXene) to significantly extend food shelf life. New photothermal materials such as MXene, due to their ultra-fast heating characteristics (such as a temperature increase of 80 °C within 60 s), can be integrated into food contact surfaces or packaging materials, which is of great significance for the quality preservation of heat-sensitive foods. MXene zinc ion-embedded agarose alginate hydrogel (MSG-Zn^2+^) demonstrates potential applications in the field of active food packaging. This hydrogel is formed by crosslinking natural polysaccharide agar (AG), alginate (SA), Ti_3_C_2_T_x_ MXene, and Zn^2+^. It has high hydrophilicity, high swelling capacity, and good biocompatibility and can efficiently absorb the exuded liquid from food surfaces, maintaining the dryness of the packaging microenvironment. For instance, the MXene hydrogel loaded with Zn^2+^ can absorb the exuded liquid from food while inhibiting the growth of spoilage bacteria through photothermal–chemical synergy, thereby extending the shelf life of fresh food [109]. Additionally, MXene films (such as Ti_3_C_2_T_x_/chitosan) can monitor the microbial activity within the packaging in real time and trigger sterilization through NIR triggering. The core advantage lies in its photothermal–chemical synergistic antibacterial properties: under 808 nm NIR irradiation, Ti_3_C_2_T_x_ MXene converts light energy into heat, eliminating food spoilage bacteria via the photothermal effect (PTT). Meanwhile, Zn^2+^ exerts chemical antibacterial effects by disrupting bacterial cell membranes, interfering with enzymatic systems, and inhibiting ATP synthesis. This dual mechanism synergistically enhances broad-spectrum antibacterial efficiency against both Gram-positive and Gram-negative bacteria [102].

#### 3.2.2. Surface Modification of Food Processing Equipment

MXene nanosheets (such as Nb_2_C) can form highly adhesive antibacterial coatings with polymer matrices (such as polyurethane or epoxy resin) through cold spraying technology. These coatings can achieve a local temperature jump (ΔT > 60 °C) within 5 min under near-infrared light (808 nm, power density 1.5 W/cm^2^) irradiation, efficiently killing foodborne pathogens bacteria on the device surface through photothermal effect. Taking the Ru@Bi_2_S_3_/Nb_2_C composite coating as an example, its unique “plasma resonance-semiconductor bandgap” synergy can enhance the light absorption efficiency (>80%), with a kill rate of 99.9% for *Listeria monocytogenes* and *Salmonella*, significantly superior to traditional sodium hypochlorite disinfection (requiring 15 min of contact and leaving residual chlorinated by-products) [103]. Such coatings have both physical barrier and photothermal self-cleaning functions: the dense-layered structure of MXene can prevent bacterial migration, and periodic NIR irradiation can achieve on-demand sterilization without chemical residues, especially suitable for in situ disinfection of difficult-to-remove equipment such as conveyor belts and cutting tools.

#### 3.2.3. Immediate Sterilization Technology

For the continuous sterilization requirements of liquid foods (fruit juice, milk, etc.), MXene-based composite materials (such as Ti_3_C_2_T_x_) can be loaded onto porous ceramics or carbon fiber carriers to construct a flow-type photocatalytic–photothermal dual-functional reactor. Its core advantages lie in energy efficiency optimization, synergy mechanism, and process compatibility: The narrow bandgap characteristic of Ti_3_C_2_T_x_ (≈1.8 eV) enables it to excite photogenerated electron–hole pairs under low-power near-infrared light (NIR, 0.5 W/cm^2^) irradiation, driving the production of ROS, while the photothermal effect raises the system temperature to 70–80 °C. The thermal energy accelerates the lipid peroxidation of bacterial membranes and disrupts protein folding, while ROS (such as ·OH, ^1^O_2_) directly damage bacterial DNA and enzyme systems, and their combined action reduces the colony count by four orders of magnitude (from 10^6^ CFU/mL to 10^2^ CFU/mL). Under a flow rate of ≤10 L/h, this system still maintains a 90% sterilization rate, and the damage rate to food color and nutrients (such as vitamin C, whey protein) is lower than that of traditional pasteurization [121]. This technology provides a low-energy (energy consumption < 0.1 kWh/L) and high-throughput non-thermal sterilization solution for thermally sensitive liquid foods.

## 4. Summary and Prospect

In this work, the application of MXene in the detection and sterilization of foodborne pathogens was systematically reviewed. Due to the high electrical conductivity, large specific surface area, tunable electronic structure, and abundant surface functional groups, MXene can be used as an ideal material for constructing highly sensitive biosensors and efficient antibacterial platforms. The paper comprehensively discusses the latest advances in MXene-based electrochemical sensors, SERS substrates, fluorescence platforms, and dual-mode sensing systems for foodborne pathogen detection. Furthermore, it elaborates on the photothermal, chemical, and physical antibacterial mechanisms of MXene in sterilization applications. Finally, practical cases of MXene in active food packaging, surface modification of equipment, and real-time sterilization technology are also highlighted.

In the field of food safety detection, MXene-based novel biosensors significantly enhance the sensitivity, selectivity, and linear range for detecting foodborne pathogens through signal amplification strategies and nanomaterial optimization. By integrating advanced molecular recognition elements and streamlined operational procedures, MXene-based sensors improve detection efficiency and field applicability, providing efficient and reliable tools for food safety monitoring with broad application prospects. Furthermore, through mechanisms such as photothermal–chemical antibacterial synergy, MXene-based nanomaterials, multifunctional hydrogels, and photocatalytic heterostructures demonstrate remarkable potential in sterilization of foodborne pathogens, offering new strategies for food safety monitoring. Despite the significant progress in MXene-based application in controlling foodborne pathogens, several challenges and opportunities remain for the further development and practical application of MXene in food safety, which also underscore current research gaps and emerging bottlenecks.

(a) Enhancing Detection Performance and Addressing Practical Application Bottlenecks: Future research should focus on optimizing the surface modification of MXene (e.g., precise regulation of functional groups such as –OH, –F, or grafting specific bio-recognition elements) to improve its target recognition capability and long-term stability in complex food matrices (such as dairy products and seafood) and reduce matrix interference. Furthermore, a critical research gap lies in the in-depth assessment of the cost analysis and economic feasibility of MXene-based sensors, with clearly defined quantitative goals including developing MXene sensors with a shelf life exceeding 6 months under ambient conditions and achieving over 99.9% sterilization efficiency within 5 min of near-infrared irradiation. Future work needs to explore the costs of large-scale production, its economic competitiveness compared to traditional detection methods (e.g., PCR, ELISA), and the economic feasibility of deployment in real-world food industry environments.

(b) Optimizing Sterilization Efficiency and Assessing Safety and Scalability: Further investigation into the synergistic mechanisms of photothermal, chemical, and physical antibacterial effects is needed—for instance, optimizing the ratio of MXene to metal ions (Zn^2+^, Ag^+^) or semiconductor materials (TiO_2_, Bi_2_S_3_) to enhance sterilization efficiency. Concurrently, biocompatibility and long-term safety assessments are paramount, especially for food contact applications, requiring a comprehensive evaluation of MXene’s potential toxicity (e.g., long-term release of metal ions). An emerging challenge is the large-scale production (scalability) of these efficient antimicrobial materials and the regulatory approval hurdles they face when moving from the laboratory to commercialization. Developing MXene composite materials that comply with food safety regulations is crucial for advancing their practical application.

(c) Integrating Intelligent Systems and Expanding Sustainable Applications: By deeply integrating MXene-based sensors with Internet of Things (IoT) and artificial intelligence (AI) technologies and developing integrated material systems with dual functions of pathogens detection and sterilization, real-time monitoring and simultaneous elimination of pathogens throughout the entire food production, transportation, and storage chain can be achieved. Further compounding MXene with biodegradable materials (e.g., chitosan, sodium alginate) enables the construction of functional materials aligned with the concept of sustainable food packaging. The development of smartphone-compatible MXene-based SERS sensors and wearable photothermal sterilization devices will promote the practical development of on-site rapid detection and on-demand sterilization technologies.

MXene has gained significant traction in the detection and sterilization of foodborne pathogens due to its unique properties. Sensors based on MXene can not only integrate internet and AI technologies to enable intelligent and rapid detection of foodborne pathogens but also collect and process data in a timely manner, providing users with immediate results. Furthermore, when combined with other materials, MXene can achieve more accurate detection and elimination of foodborne pathogens even in complex matrices. As a result, MXene has enabled more precise, intelligent, and sensitive applications in the detection and sterilization of foodborne pathogens, encouraging further development of MXene materials and enabling progress in MXene-related technologies.

## Data Availability

The original contributions presented in this study are included in the article. Further inquiries can be directed to the corresponding authors.
